# Plasma bioactive adrenomedullin predicts outcome after acute stroke in early rehabilitation

**DOI:** 10.1038/s41598-023-30633-9

**Published:** 2023-03-24

**Authors:** Azadeh Shafieesabet, Nadja Jauert, Oliver Hartmann, Birte Arlt, Michael Joebges, Wolfram Doehner

**Affiliations:** 1 Berlin Institute of Health at Charité – Universitätsmedizin Berlin, BIH Center for Regenerative Therapies (BCRT), Berlin, Germany; 2 Department of Cardiology (Virchow Klinikum), Charité Universitätsmedizin Berlin and German Centre for Cardiovascular Research (DZHK), Partner Site Berlin, Berlin, Germany; 3 Center for Stroke Research Berlin (CSB), Charité-Universitätsmedizin Berlin, Berlin, Germany; 4 SphingoTec GmbH, Hennigsdorf, Germany; 5 Department of Neurology, Brandenburg Klinik, Bernau and Kliniken Schmieder, Konstanz, Germany

**Keywords:** Predictive markers, Prognostic markers

## Abstract

An early and reliable prediction of outcomes after stroke is important for early effective stroke management and the adequate optimal planning of post-stroke rehabilitation and long-term care. Bioactive adrenomedullin (bio-ADM) is a 52-amino acid peptide that is an important peptide hormone in nervous system diseases. The aim of this study was to investigate the prognostic value of bio-ADM on outcomes after rehabilitation in patients with stroke. A total of 557 consecutive patients with a primary diagnosis of ischemic or hemorrhagic stroke (age 69.6–12.9 years, male 51.3%, ischemic stroke 72.5%), who were admitted to an in-patient early rehabilitation center directly after discharge from acute stroke hospital care, were enrolled in this prospective observational study. Plasma concentrations of bio-ADM were determined by using a chemiluminescence immunoassay (functional assay sensitivity 8 pg/ml). The early rehabilitation barthel index (ERBI) was used for the neurological assessment of the patients. The plasma bio-ADM level was analyzed in association with 6-month all-cause mortality as well as a composite outcome of all-cause mortality, unscheduled re-hospitalization, or transfer to a long-term care facility in a vegetative or minimally conscious state. Bio-ADM levels significantly increased in patients with ischemic stroke who died compared to surviving patients (40.4 pg/ml vs. 23.8 pg/ml, p < 0.001) or in those with composite outcomes compared to those with no events (36.9 pg/ml vs. 23.5 pg/ml, p < 0.001). Six-month all-cause mortality was higher in all patients with bio-ADM levels > 70 pg/ml (HR 4.83 [CI 2.28–10.2]). Patients with bio-ADM levels > 70 pg/ml also had higher rates of 6-month composite outcomes (HR 3.82 [CI 2.08–7.01]). Bio-ADM was an independent predictor of all-cause mortality and 6-month composite outcomes after adjusting for age, gender, and ERBI (adjusted OR 1.5; 95% CI 1.0–2.1; p = 0.047 and adjusted OR 1.48; 95% CI 1.1–2.0; p = 0.01, respectively). Bio-ADM may be a suitable novel biomarker to assess the outcomes of patients in rehabilitation after acute stroke. Elevated bio-ADM concentrations may have prognostic value for fatal and nonfatal events in patients with ischemic stroke during early rehabilitation.

## Introduction

Stroke from a global perspective is the second largest cause of death and also the second most common cause of adult disability-adjusted life years (DALYs) in our society^1^. Early rehabilitation interventions after stroke can enhance post-stroke functional recovery and minimize long-term disability^2^. An early and reliable prognosis for recovery after acute stroke is important for stroke management and also for the optimal planning of post-stroke care^3^. Biomarkers as prediction tools could be used by clinicians to improve the accuracy of prognoses and escalate rehabilitation programs^4^.

Adrenomedullin is a 52-amino acid peptide with vasoactive properties and expressed in numerous tissues, including blood vessels, skeletal muscles, heart, lungs, and nerve tissue^5^. Adrenomedullin exerts multiple physiological functions with possible clinical importance and, in recent years, has been shown as a potential prognostic biomarker in different metabolic conditions such as dyspnea^6^, heart failure^7^, cardiogenic shock^8^, and sepsis^9^.

Circulating biologically active adrenomedullin (bio-ADM) is a promising novel biomarker that is an important regulatory factor in nervous system diseases. Bio-ADM has been demonstrated to have a facilitating or neuroprotective effect against brain injury, including hemorrhagic or ischemic stroke and traumatic brain injury^10^. Bio-ADM also has strong anti-inflammatory activities and acts through different receptors to modulate vascular integrity. It helps to maintain vascular structure and regulates angiogenesis, acting as a vasoprotector against vascular injury. These effects are observed in both acute and chronic cerebral ischemia^11–13^. Furthermore, adrenomedullin can act as a regulator to promote nerve regeneration in pathological conditions. Therefore, adrenomedullin is an important participant in nervous system diseases^10^.

Adrenomedullin expression is upregulated by hypoxia through the activation of the hypoxia inducible factor-1 (HIF-1) pathway^14^. Previous studies have demonstrated that adrenomedullin levels in blood are closely associated with the severity and clinical outcomes of patients with acute ischemic stroke^15,16^ as well as other types of neurological damage, including traumatic brain injury^17^ or intracerebral hemorrhage^18–20^. All these studies evaluated the elevated levels of bio-ADM in acute phase of stroke, but the evolution of those levels during post-acute phase was not studied.

The aim of this clinical study was to further investigate the prognostic values of bio-ADM on outcomes of rehabilitation in stroke patients. Our hypothesis was that elevated level of bio-ADM may predict the unfavorable outcomes in the rehabilitation setting.

## Methods

### Study population and enrollment criteria

A total of 557 consecutive patients with a primary diagnosis of stroke who were admitted to the Berlin-Brandenburg rehabilitation center for comprehensive in-patient stroke rehabilitation were enrolled in this prospective study.

The subjects were more than 18 years of age, and each had an ischemic or hemorrhagic stroke. Stroke is defined as an acute onset of focal or global neurological findings dysfunction lasting more than 24 h, confirmed by both clinical and radiographic means^21^. All the patients were admitted to an in-patient early rehabilitation center directly after hospital discharge.

For the neurological assessment of the patients, the Early Rehabilitation Barthel Index (ERBI) was used. ERBI values were classified into four different categories based on the German ICD-10 catalogue^22^: “total” dependency, ranging from − 325 to − 201; “severe” dependency, ranging from − 200 to − 76; “moderate” dependency, ranging from − 75 to 30; and “slight” dependency, ranging from 31 to 100.

### Blood sampling

Venous blood was collected from patients on the first day of admission to the rehabilitation center and placed in tubes with ethylenediaminetetraacetic acid (EDTA) as an anticoagulant. The samples were immediately centrifuged, aliquoted, and stored at − 80 °C until batch analysis. Routine biochemical parameters were assessed in the standard clinical laboratory.

### Measurement of bio-ADM plasma level concentrations

The plasma bio-ADM levels were determined through a chemiluminescence sandwich immunoassay, provided by SphingoTec GmbH, as described previously^23^. Briefly, two mouse monoclonal antibodies were used. The solid phase antibody, coated to white polystyrene microtiter plates, was directed against the middle region of ADM, while the capture antibody was labeled with acridinium NHS-ester and directed against the amidated C-terminal moiety of ADM. The functional assay sensitivity was 8 pg/ml.

### Study endpoints

The patients were followed up until death or discharge from the rehabilitation center. The patients were discharged from the rehabilitation center either as readmission to a primary hospital because of clinical deterioration, transfer to a long-term care facility in a vegetative or minimally conscious state, discharge to a nursing home after completion of the rehabilitation program, or discharge for continuing care at home. The endpoints selected for these analyses were all-cause mortality and a composite outcome of all-cause mortality, readmission to a primary hospital, or transfer to a long-term care facility in a vegetative or minimally conscious state.

### Statistics

The values are expressed as medians and interquartile ranges (IQR) or counts and percentages, as appropriate. Group comparisons of continuous variables were performed using the Kruskal–Wallis test. For comparisons involving more than 2 categories, corresponding post-hoc tests were applied, with pairwise comparisons adjusted appropriately for multiple comparisons. Categorical data was compared using Pearson’s chi-squared test for count data.

Logistic regression was used to analyze the association between bio-ADM and the study endpoints. Bio-ADM was adjusted by age, gender, and ERBI, and the added value of bio-ADM was evaluated based on the likelihood ratio chi-square test for nested models. Bio-ADM data was log-transformed. Receiver operating characteristic (ROC) curves were constructed for illustration and area under the curve (AUC) determined. For multivariable models, the AUCs were bootstrap corrected. For continuous variables, odds ratios (OR) were standardized to describe the OR for a change of one IQR.

A previously reported cut-off value of 70 pg/ml^24,25^, was applied in Kaplan–Meier curves for illustration. For Kaplan–Meier plots, patients discharged to home or a nursing home were treated as alive at 180 days of follow-up, while follow-up was not extended for patients transferred to a long-term care facility in a vegetative or minimally conscious state or readmitted to a primary hospital because of clinical deterioration.

All statistical tests were two-tailed, and a two-sided p-value of 0.05 was considered for significance. The statistical analyses were performed using R version 3.4.3 (http://www.r-project.org) and Statistical Package for the Social Sciences (SPSS) version 22.0 (SPSS Inc., Chicago, Illinois, USA).

### Ethics approval and consent to participate

This protocol was approved by the local Ethics Committee of the Brandenburg Medical Association, and written informed consent was obtained from the patients included in this study. All methods were performed in accordance with the relevant guidelines and regulations.

## Results

### Clinical characteristics

The main clinical characteristics of the study population are shown in Table 1. In the study, 557 patients were included in an in-patient early rehabilitation center directly after hospital discharge, 286 (51.3%) men and 271 (48.7%) women with a mean age of 69.6 years (SD = 12.9). Among all the patients, 404 (72.5%) presented with ischemic stroke and 153 (27.5%) with hemorrhagic events. The average interval between stroke onset and admission to the rehabilitation center was 25.8 (SD = 22.1) days in our study population. During a median follow-up of six [0.5–11] months, 38 (6.8%) patients died, 26 (4.7%) were readmitted to a primary hospital because of clinical deterioration, and 3 (0.5%) were referred to a long-term care facility in a vegetative or minimally conscious state.

**Table 1:** Patients’ characteristics and 6-month clinical outcomes.

Characteristics	Overall	6-Month all-cause mortality		6-Month composite outcome			
		Non-survival	Survival	p-value	Composite outcome	No event	p-value
Age-median [IQR]	72 [61 to 79]	74.5 [71 to 83.5]	72 [61 to 79]	0.028	72.5 [61 to 80]	72 [61 to 79]	n.s.
Gender, male n (%)	286 (51.3)	18 (47.4)	268 (51.6)	n.s.	32 (47.8)	254 (51.8)	n.s.
Stroke type, ischemic n (%)	404 (72.5)	26 (68.4)	378 (72.8)	n.s.	44 (65.7)	360 (73.5)	n.s.
AF, n (%)	192 (34.5)	16 (42.1)	176 (33.9)	n.s.	27 (40.3)	165 (33.7)	n.s.
DM, n (%)	176 (31.6)	15 (39.5)	161 (31)	n.s.	22 (32.8)	154 (31.5)	n.s.
HTN, n (%)	432 (77.6)	31 (81.6)	401 (77.3)	n.s.	48 (71.6)	384 (78.5)	n.s.
HLP, n (%)	134 (24.1)	6 (15.8)	128 (24.7)	n.s.	9 (13.4)	125 (25.6)	0.03
CVD, n (%)	140 (25.1)	15 (39.5)	125 (24.1)	n.s.	23 (34.3)	117 (23.9)	n.s.
HF, n (%)	47 (8.4)	6 (15.8)	41 (7.9)	n.s.	12 (17.9)	35 (7.1)	0.008
CKD, n (%)	99 (17.8)	9 (23.7)	90 (17.3)	n.s.	18 (26.9)	81 (16.5)	n.s.
MI-median [IQR]	103 [14.75 to 136.75]	0 [0 to 38]	107 [21.5 to 139]	< 0.001	0 [0 to 99.5]	107 [31 to 140]	< 0.001
TCT-median [IQR]	49 [12 to 87]	0 [0 to 24]	61 [12 to 87]	< 0.001	6 [0 to 48]	61 [12 to 87]	< 0.001
ERBI-median [IQR]	15 [− 45 to 45]	− 45 [− 100 to 0]	20 [− 40 to 45]	< 0.001	− 37.5 [− 100 to 5]	20 [− 38.75 to 45]	< 0.001
Cr-median [IQR]	71 [57 to 93.5]	67 [56 to 106]	71.5 [58 to 93]	n.s.	72 [55.5 to 112.25]	71 [57.5 to 93]	n.s.
GFR-median [IQR]	89 [63.25 to 112.75]	88 [60 to 126.5]	89 [64 to 112]	n.s.	88 [58 to 122]	89 [64 to 112]	n.s.
Total protein-median [IQR]	64.4 [59.8 to 68.9]	57.8 [53.6 to 64.1]	64.75 [60.58 to 69.2]	< 0.001	59.5 [53.95 to 65.15]	64.85 [60.9 to 69.2]	< 0.001
Hb-median [IQR]	7.8 [6.8 to 8.6]	6.9 [6.2 to 7.9]	7.8 [6.8 to 8.6]	< 0.001	6.9 [6.1 to 8]	7.9 [6.9 to 8.7]	0.001
WBC-median [IQR]	8 [6.4 to 10.2]	9.65 [8.1 to 11.75]	7.9 [6.4 to 10.1]	0.0012	8.6 [7.2 to 11.2]	7.9 [6.4 to 10.1]	0.001
CRP-median [IQR]	13 [5.35 to 33.6]	42.5 [19.23 to 103.03]	12.45 [4.68 to 28.65]	< 0.001	32.1 [8.25 to 81.55]	12.4 [4.4 to 28.4]	< 0.001
GGT-median [IQR]	0.96 [0.52 to 1.81]	1.21 [0.81 to 1.97]	0.95 [0.51 to 1.79]	0.041	1.31 [0.73 to 2.29]	0.93 [0.5 to 1.71]	0.04
GPT-median [IQR]	0.47 [0.32 to 0.72]	0.58 [0.35 to 1.02]	0.46 [0.32 to 0.71]	n.s.	0.54 [0.33 to 0.86]	0.46 [0.32 to 0.7]	n.s.
Anticoagulation therapy, n (%)	350 (62.8)	12 (70.6)	338 (71)	n.s.	33 (80.5)	317 (70.1)	n.s.

*AF* atrial fibrillation, *DM* diabetes mellitus, *HTN* hypertension, *HLP* hyperlipidemia, *CVD* chronic vascular disease, *HF* heart failure, *CKD* chronic kidney disease, *MI* motricity index, *TCT* trunk control test, *ERBI* early rehabilitation barthel index, *Cr* creatinine, *GFR* glomerular filtration rate, *Hb* hemoglobin, *WBC* white blood count, *CRP* C-reactive protein, *GGT* gamma-glutamyl transferase, *GPT* glutamic-pyruvic transaminase.

### Plasma bio-ADM

**Figure 1:**
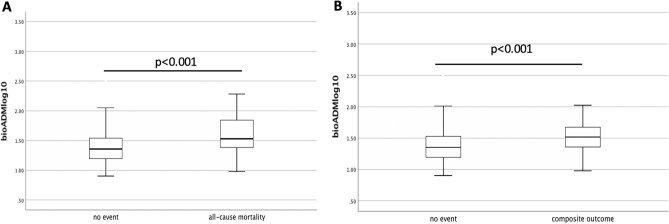
Association of bio-ADM levels with outcome of all-cause mortality (**A**) and composite outcome (**B**) in all patients with stroke (death endpoint, p < 0.001/composite endpoint, p < 0.001).

The median concentration of bio-ADM at the baseline was 23.7 [IQR: 15.9–36.3] pg/ml. Bio-ADM levels significantly increased in patients with the endpoint of all-cause mortality (median [IQR]: 33.8 pg/ml [24.1 to 70.1] vs. 22.8 pg/ml [15.7 to 34.7]) or composite outcome (median [IQR]: 32.9 pg/ml [22.2 to 48.5] vs. 22.5 pg/ml [15.5 to 33.8]) compared to those with no event (each group vs. no event, p < 0.001) (Fig. 1).

Bio-ADM did not show significant differences between stroke subtypes (ischemic stroke: 24.8 pg/ml [16.6 to 36.9]; and hemorrhagic stroke: 21.7 pg/ml [14.4 to 31.3]; p > 0.05).

**Figure 2:**
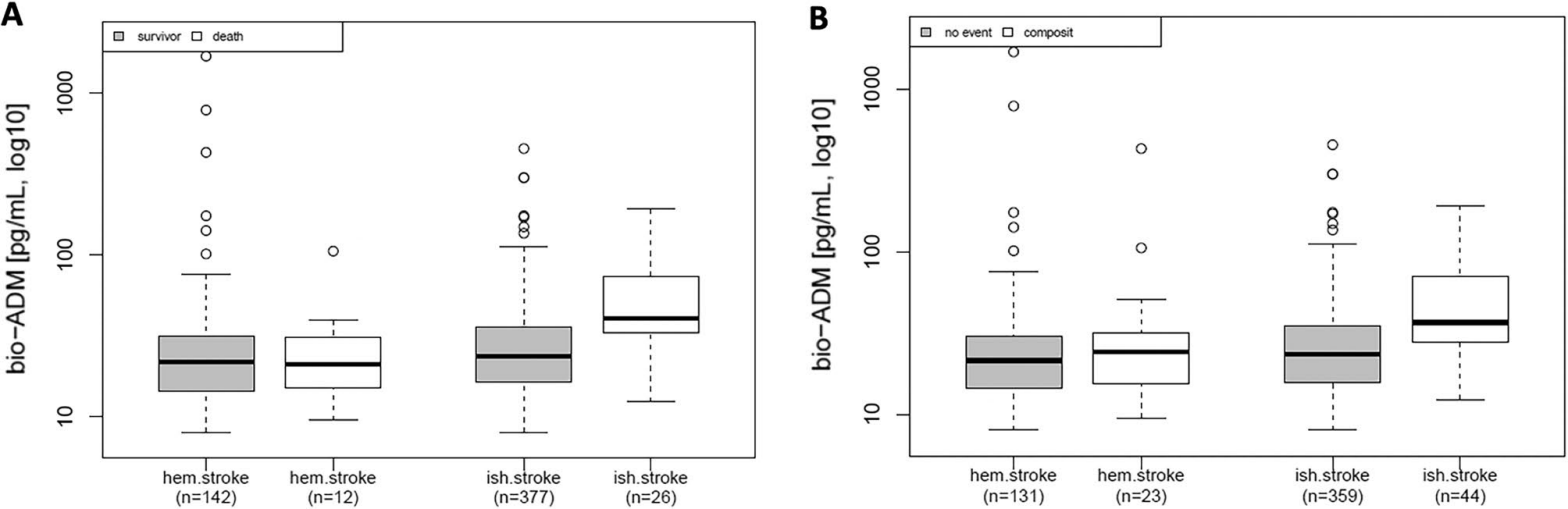
Association of bio-ADM levels with outcome of all-cause mortality (**A**) and composite outcome (**B**) in patients with hemorrhagic or ischemic stroke diagnosis. (Ischemic stroke: death endpoint, p < 0.001/composite endpoint, p < 0.001; Hemorrhagic stroke: death endpoint, p = 0.94/composite endpoint, p = 0.36).

An analysis of the study endpoints according to stroke subtypes revealed that the patients with ischemic stroke had significantly increased bio-ADM in the all-cause mortality (median [IQR]: 40.4 pg/ml [33.1 to 75.7] vs. 23.8 pg/ml [16.2 to 36]) or composite outcome (median [IQR]: 36.9 pg/ml [27.2 to 70.7] vs. 23.5 pg/ml [15.8 to 35]) group compared to those with no event (each group vs. no event, p < 0.001). However, in patients with hemorrhagic stroke in either the all-cause mortality (median [IQR]: 21.3 pg/ml [14.6 to 31.7] vs. 21.7 pg/ml [14.4 to 31.3]) or composite outcome (median [IQR]: 24.2 pg/ml [15.2 to 32.4] vs. 21.5 pg/ml [14.1 to 30.1]) group, no significant differences were observed compared to those with no event (each group vs. no event, nonsignificant) (Fig. 2).

Higher levels of bio-ADM were observed in patients with diabetes mellitus (median [IQR]: 29.6 pg/ml [18.5 to 41.7] vs. 22.4 pg/ml [15.4 to 33.2]), atrial fibrillation (median [IQR] 28.5 pg/ml [20.1 to 44.3] vs. 21.7 pg/ml [14.4 to 33.1]), cardiovascular disease (median [IQR]: 28.1 pg/ml [20.2 to 44.9] vs. 22.4 pg/ml [15.1 to 33.5]), or chronic kidney disease (median [IQR] 34.9 pg/ml [22.5 to 47.1] vs. 21.9 pg/ml [15.4 to 33.3]) compared to those with no comorbidities (p < 0.001) (Fig. S1).

**Figure 3:**
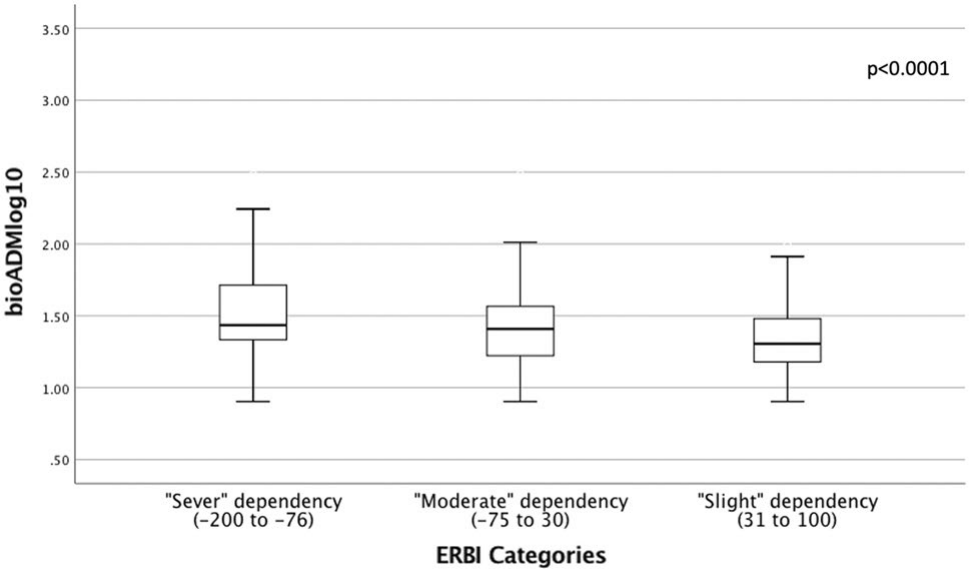
Bio-ADM levels at admission of patients, grouped by the Early Rehabilitation Barthel Index (ERBI) (p < 0.001). Categories of the ERBI according to ICD-10-German Modification^21^: Slight dependency [31 to 100], Moderate dependency [− 75 to 30 points], Sever dependency [− 200 to − 76 points], Total dependency [− 201 to − 325]. There were no patients in Total dependency category. Post-hoc comparisons showed significant differences between groups “slight dependency” and “moderate dependency” and between “slight dependency” and “sever dependency” (both p < 0.05).

Plasma bio-ADM concentrations also varied significantly by ERBI category^21^, being the highest in patients in the lowest functional capacity (median [IQR]: 27.2 pg/ml [21.4 to 53.8] in “severe” dependency, 25.6 pg/ml [16.7 to 36.8] in “moderate” dependency, and 20.2 pg/ml [15 to 30.3] in “slight” dependency) (ANOVA, p < 0.001) Post-hoc comparisons showed significant differences between groups “slight dependency” and “moderate dependency” and between “slight dependency” and “sever dependency” (both p < 0.05) (Fig. 3).

### Bio-ADM and 6-month all-cause mortality

#### All stroke patients

**Figure 4:**
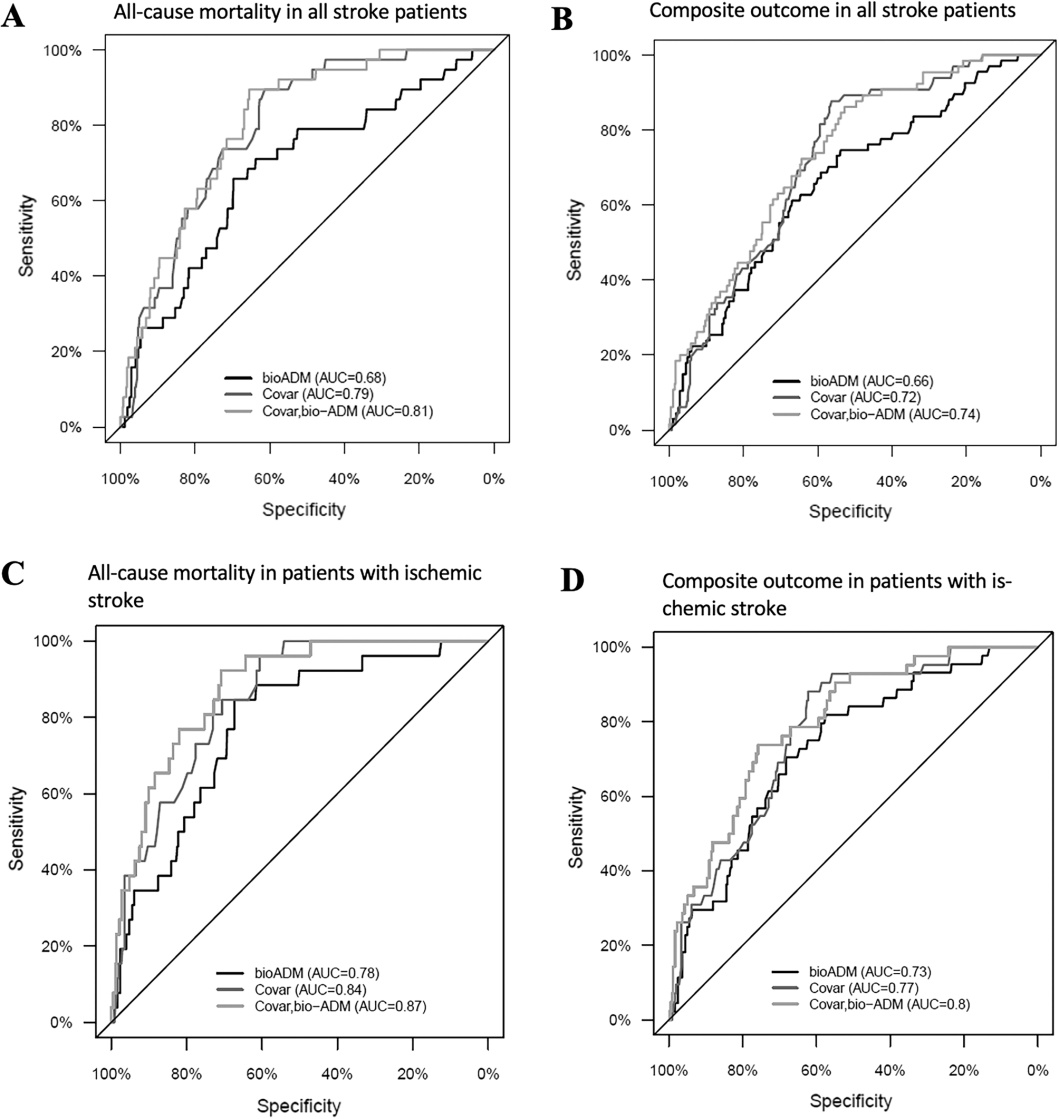
Receiver-operating characteristic plot for bio-ADM, covariables (age, gender, ERBI) and the combination of the two in all stroke patients with all-cause mortality (**A**) and with composite outcome (**B**) and in patients with ischemic stroke and all-cause mortality (**C**) and with composite outcome (**D**). Multivariable AUC given here not bootstrap corrected.

**Figure 5:**
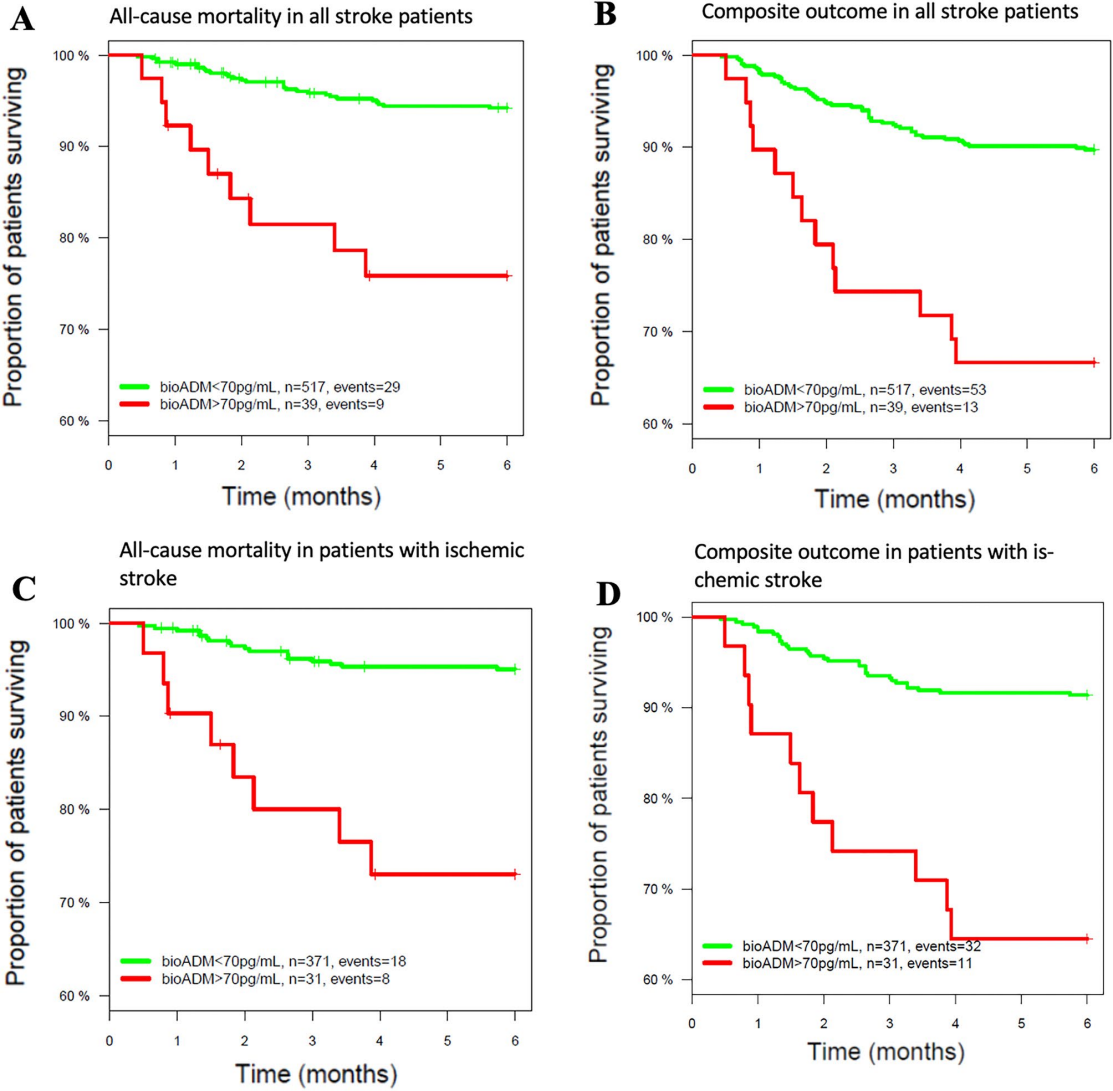
Kaplan–Meier plots for all stroke patients with high bio-ADM levels (> 70 pg/ml) versus low bio-ADM levels (< 70 pg/ml) for all-cause mortality (**A**) or composite outcome (**B**) and in patients with ischemic stroke and high bio-ADM levels (> 70 pg/ml) versus low bio-ADM levels (< 70 pg/ml) for all-cause mortality (**C**) or composite outcome (**D**). Hazard ratio for all stroke patients with all-cause mortality is 4.83 [CI 2.28–10.2] and with composite outcome is 3.82 [CI 2.08–7.01]. One patient had missing follow up time and is therefore excluded for this figure. Hazard ratio in patients with ischemic stroke for all-cause mortality is 6.24 [CI 2.71–14.4] and for composite outcome is 4.86 [CI 2.45–9.64]. For n = 1 patient time to event was unknown.

Six-month all-cause mortality was 6.8% (38/557). As shown in Fig. 4A, in ROC curve analysis, the plasma bio-ADM level predicted 6-month all-cause mortality with an AUC of 0.68 (95% CI 0.59–0.77, p < 0.001). When using a cut-off for bio-ADM levels > 70, the risk of all-cause mortality was significantly higher (HR 4.83, 95% CI [2.2.8–10.2]; Fig. 5A).

A multivariate logistic regression analysis showed that adding age, gender, and ERBI to a model including bio-ADM improved the bootstrap-corrected AUC from 0.78 to 0.79 (adjusted, standardized OR for bio-ADM: 1.5; 95% CI 1.0–2.1; p = 0.047). Therefore, bio-ADM was an independent predictor of six-month all-cause mortality after adjusting for age, gender, and ERBI (p = 0.048 for added value).

#### Patients with ischemic stroke

Six-month all-cause mortality was 6.5% (26/403) in patients with ischemic stroke. As shown in Fig. 4C, in ROC curve analysis, the plasma bio-ADM level predicted 6-month all-cause mortality with an AUC of 0.78 (95% CI 0.69–0.86, p < 0.001). When using a cut-off for bio-ADM levels > 70, the risk of all-cause mortality was significantly higher (HR 6.24, 95% CI [2.71–14.4]; Fig. 5C).

A multivariate logistic regression analysis showed that adding age, gender, and ERBI to a model including bio-ADM improved the bootstrap-corrected AUC from 0.84 to 0.86 (adjusted, standardized OR for bio-ADM: 2.1; 95% CI 1.3–3.4; p = 0.003). Therefore, bio-ADM was an independent predictor of 6-month all-cause mortality after adjusting for age, gender, and ERBI in patients with ischemic stroke (p = 0.004 for added value).

### Bio-ADM and 6-month composite outcome

#### All stroke patients

Of all the patients, 67 (12%) had experienced composite outcomes in 6 months. As shown in Fig. 4B, in ROC curve analysis, the plasma bio-ADM levels predicted 6-month composite outcomes with an AUC of 0.66 (95% CI 0.59–0.73, p < 0.001). The patients with bio-ADM levels > 70 pg/ml had higher rates of six-month composite outcomes (HR 3.82, 95% CI [2.08–7.01]; Fig. 5B).

A multivariate logistic regression analysis showed that adding age, gender, and ERBI to a model including bio-ADM improved the bootstrap-corrected AUC from 0.71 to 0.72 (adjusted, standardized OR for bio-ADM 1.48; 95% CI 1.1–2.0; p = 0.01). Therefore, bio-ADM was an independent predictor of 6-month composite outcome after adjusting for age, gender, and ERBI (p = 0.01 for added value).

#### Patients with ischemic stroke

Of the patients with ischemic stroke, 44 (10.9%) had experienced composite outcomes in 6 months.

As shown in Fig. 4D, in ROC curve analysis, the plasma bio-ADM levels predicted 6-month composite outcomes with an AUC of 0.73 (95% CI 0.66–0.80, p < 0.001). The patients with ischemic stroke and bio-ADM levels > 70 pg/ml had higher rates of 6-month composite outcomes (HR 4.86, 95% CI [2.45–9.64]; Fig. 5D).

A multivariate logistic regression analysis showed that adding age, gender, and ERBI to a model including bio-ADM improved the bootstrap-corrected AUC from 0.76 to 0.79 (adjusted, standardized OR for bio-ADM 1.79; 95% CI 1.2–2.6; p = 0.004). Therefore, bio-ADM was an independent predictor of 6-month composite outcome after adjusting for age, gender, and ERBI in patients with ischemic stroke (p = 0.004 for added value).

## Discussion

The main findings of this study are that plasma concentrations of bio-ADM are elevated in stroke patients in relation to adverse clinical outcomes in terms of all-cause mortality as well as composite outcomes of mortality, vegetative or minimally conscious states, and clinical deterioration requiring immediate hospital readmission. The elevation of bio-ADM is also correlated with comorbidities as well as the severity of functional disability in post-stroke and early rehabilitation as assessed via ERBI. Bio-ADM levels > 70 pg/ml predicted six-month all-cause mortality and the composite endpoint of clinical deterioration after adjustments for age, gender, and ERBI. This is the first study to evaluate the impact of bio-ADM on the outcome of rehabilitation after acute stroke.

The clinical significance of adrenomedullin in brain injury has been emphasized in recent years. The level of plasma adrenomedullin significantly increases in acute ischemic stroke and has been found as an independent predictor of six-month outcomes in these patients^15,16^. Some clinical studies have demonstrated that elevated bio-ADM levels improve the prediction of functional outcomes in acute stroke^18,26,27^. It has also been indicated that bio-ADM plasma levels are significantly associated with mortality in patients with subarachnoid hemorrhage^28,29^ and traumatic brain injury^17^.

Adrenomedullin has been shown to play a role in brain injury, such as hemorrhagic^18,19^ and ischemic stroke^15,16^ or traumatic brain injury^17^. In pathological settings, adrenomedullin may also function as a regulator to facilitate nerve regeneration. As a result, adrenomedullin is a key player in nervous system disorders^10^. These findings provide preliminary evidence of the importance of adrenomedullin in neurological conditions.

The results of our study identified bio-ADM as an independent predictor of clinical outcomes in post-acute stroke. The ROC curve showed that plasma bio-ADM concentration on admission to post-stroke rehabilitation center could noticeably predict all-cause mortality and composite outcomes. Therefore, the determination of bio-ADM in the plasma at the onset of post-stroke rehabilitation may provide the ability to identify patients at risk of 6-month all-cause mortality, readmission to a primary hospital, or transfer to a long-term care facility in a vegetative or minimally conscious state.

The function of adrenomodulin in brain injury is intricate and debated, with reports of both favorable and adverse effects. The relationship between bio-ADM levels and prognosis in stroke patients is not well established in the literature. It is counterintuitive to find higher levels of bio-ADM in patients with poor prognosis, as it is thought to be expressed as a protective peptide. However, recent findings suggest that bio-ADM may have different impacts on various regions of the brain, and its overall effects on brain injury may be a result of the collective action of these regions^10,30,31^. This could explain why higher levels of bio-ADM have been associated with a poorer prognosis in some cases, including our study.

Analysis of subgroups of stroke etiology showed that patients with ischemic stroke had significantly higher bio-ADM levels in the all-cause mortality or composite outcome group, compared to those without any events. However, no significant differences were seen in patients with hemorrhagic stroke. This discrepancy indicates that bio-ADM may play different roles in the physiopathology of ischemic and hemorrhagic strokes. The precise effect of bio-ADM in stroke recovery is complex, and the available literature provides partially conflicting results, likely due to the intricate nature of the system. It is possible that different biomarkers may be needed to address individual types of stroke.

In this study, a close relationship was found between plasma bio-ADM concentrations and ERBI, suggesting that plasma bio-ADM concentrations should reflect the severity of functional disability in post-stroke and early rehabilitation. These findings support the mechanistic role of ADM in cerebral injury given the potential role of adrenomedullin receptor genes in stroke and vascular fragility^11^.

Biomarkers are continuously investigated as important tools to predict outcomes in critical patients so as to improve resource allocation and to assist in clinical decision making^32^. In terms of stroke, biomarker development was found to be challenging for determining the diagnosis and prognosis of stroke as well as predicting poststroke recovery^33^. Therefore, the development of fast and easy-to-apply tests for evaluating bio-ADM levels may be useful for predicting patient outcomes as well as developing best-practice rehabilitation strategies to create an effective recovery program after stroke. The combination of biomarkers and clinical judgment provides clinically useful information while planning the personalized rehabilitation of a patient^34^.

The strengths of this study lie in the sample size, the prospective design, investigation of the predictive value of a biomarker in the long-term clinical course and the addition of a novel biomarker. This study is limited by the range of biomarkers as we did not pursue a multi-marker approach to identify a promising candidate out of a range of variables. Additionally, we recorded bio-ADM upon admission to the rehabilitation center on the first day. Despite all patients being admitted to the rehabilitation center immediately following hospital discharge, the time gap between the stroke onset and bio-ADM measurement was not taken into consideration in our study. Another limitation of this study is that the measurement of the bio-ADM was conducted only once, on the first day of admission to the rehabilitation center, and was not repeated multiple times. As a result, we are unable to determine the dynamic changes in the plasma level of bio-ADM during the admission period in rehabilitation center.

## Conclusions

In conclusion, bio-ADM may be a novel biomarker to assess outcomes after stroke and also to identify patients with increased risk requiring greater medical attention during rehabilitation. Elevated bio-ADM concentrations may have prognostic value for fatal and nonfatal events in patients with ischemic stroke during early rehabilitation. Our findings suggest that bio-ADM might be used to guide the management of stroke patients and possibly assess the risk of mortality or (re)hospitalization during rehabilitation. Further studies in which bio-ADM is assessed at multiple time points during post-stroke rehabilitation could help shed more light on the role of this biomarker.

## Supplementary Information

Supplementary Figure S1.

## Data Availability

The datasets used and/or analysed during the current study are available from the corresponding author on reasonable request.

## Abbreviations

Bio-ADM: Bioactive adrenomedullin
ERBI: Early rehabilitation barthel index
DALYs: Disability-adjusted life years
HIF-1: Hypoxia inducible factor-1
IQR: Interquartile range
ROC: Receiver operating characteristic
AUC: Area under the curve
OR: Odds ratio
SPSS: Statistical Package for the Social Sciences
