# Supplementary material for: Plasma bioactive adrenomedullin predicts outcome after acute stroke in early rehabilitation
Source: Sci Rep. 2023 Mar 24;13:4873. doi: 10.1038/s41598-023-30633-9 (PMC10039005; doi:10.1038/s41598-023-30633-9)
Supplement: Supplementary file 1 — Supplementary Figure S1. [file 41598_2023_30633_MOESM1_ESM.docx]

Supplement:

5


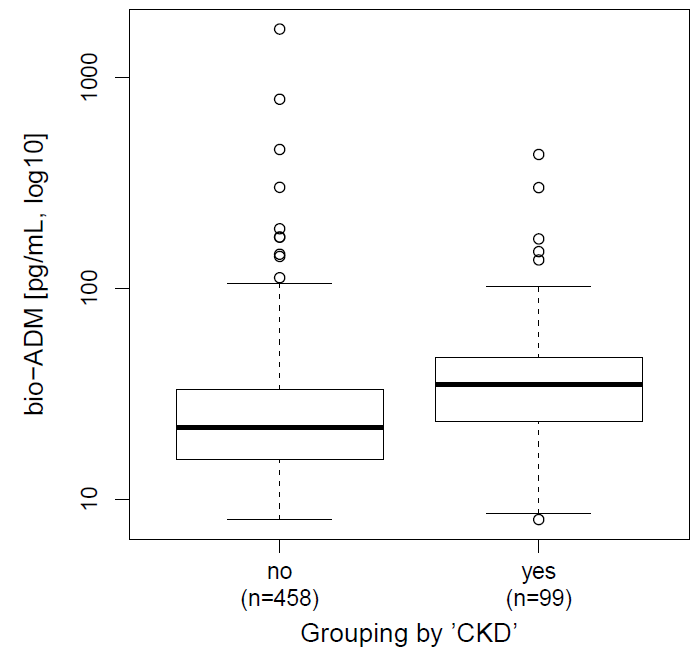

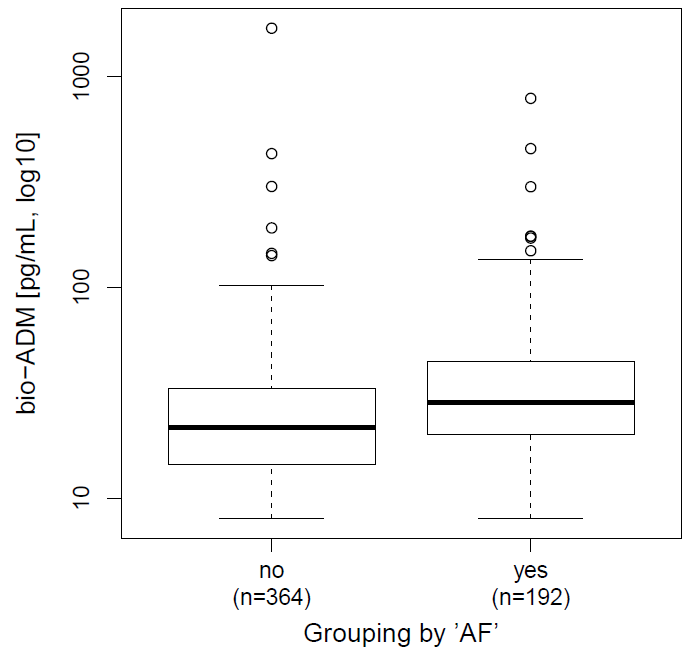


Atrial Fibrillation

Chronic Kidney Disease

Cardiovascular Disease

Diabetes Mellitus


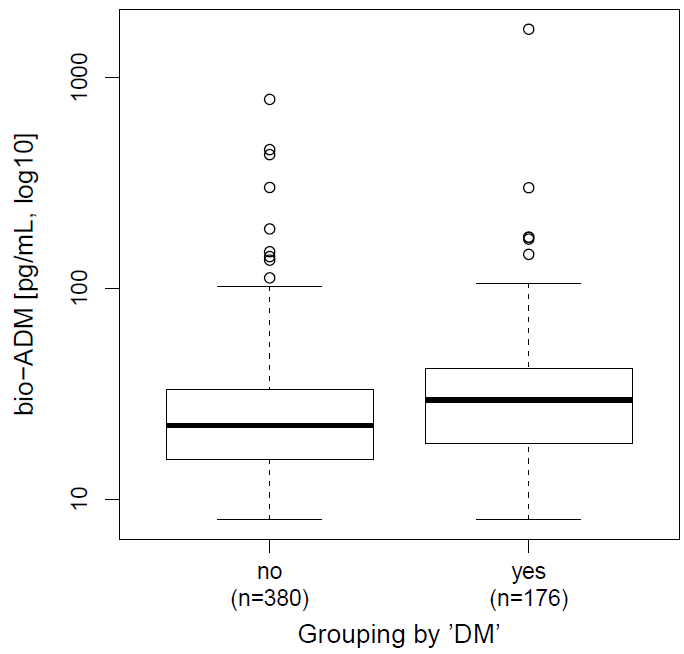

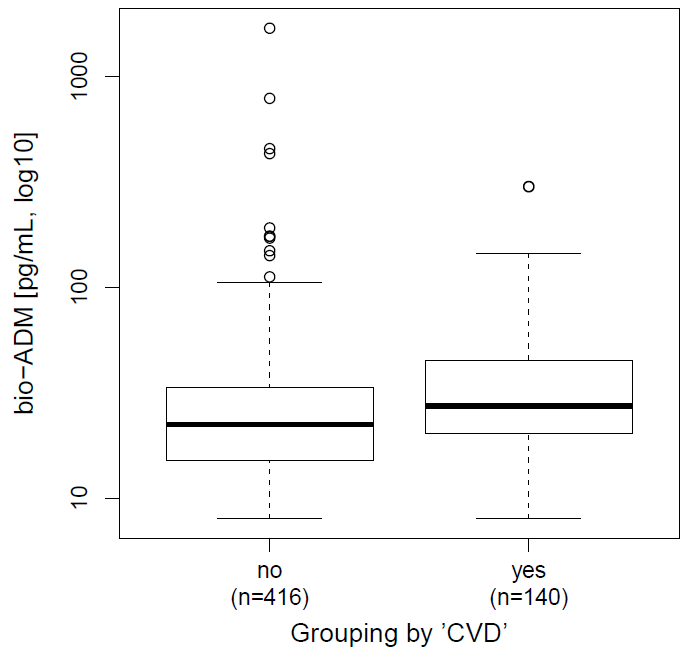


Diabetes mellitus

Cardiovascular disease

**Fig S1:** bio-ADM levels in all stroke patients with and without atrial fibrillation (p<0.0001), chronic kidney disease (p<0.0001), diabetes mellitus (p=0.0001) and cardiovascular disease (p<0.0001).
